# Measures of overnight oxygen saturation to characterize sleep apnea severity and predict postoperative respiratory depression

**DOI:** 10.1186/s12938-024-01254-8

**Published:** 2024-07-08

**Authors:** Atousa Assadi, Frances Chung, Azadeh Yadollahi

**Affiliations:** 1 KITE-Toronto Rehabilitation Institute, University Health Network, Toronto, Canada; 2 Institute of Biomedical Engineering, University of Toronto, Toronto, Canada; 3 Temerty Center for AI Research and Education in Medicine, University of Toronto, Toronto, Canada; 4 Department of Anesthesia and Pain Medicine, Toronto Western Hospital, University Health Network, University of Toronto, Toronto, Canada; 5 Institute of Medical Science, Temerty Faculty of Medicine, University of Toronto, Toronto, Canada

**Keywords:** Sleep apnea, Oxyhemoglobin saturation (SpO_2_), Postoperative respiratory depression, Prediction, Signal processing, Logistic regression

## Abstract

**Background:**

Sleep apnea syndrome, characterized by recurrent cessation (apnea) or reduction (hypopnea) of breathing during sleep, is a major risk factor for postoperative respiratory depression. Challenges in sleep apnea assessment have led to the proposal of alternative metrics derived from oxyhemoglobin saturation (SpO_2_), such as oxygen desaturation index (ODI) and percentage of cumulative sleep time spent with SpO_2_ below 90% (CT90), as predictors of postoperative respiratory depression. However, their performance has been limited with area under the curve of 0.60 for ODI and 0.59 for CT90. Our objective was to propose novel features from preoperative overnight SpO_2_ which are correlated with sleep apnea severity and predictive of postoperative respiratory depression.

**Methods:**

Preoperative SpO_2_ signals from 235 surgical patients were retrospectively analyzed to derive seven features to characterize the sleep apnea severity. The features included entropy and standard deviation of SpO_2_ signal; below average burden characterizing the area under the average SpO_2_; average, standard deviation, and entropy of desaturation burdens; and overall nocturnal desaturation burden. The association between the extracted features and sleep apnea severity was assessed using Pearson correlation analysis. Logistic regression was employed to evaluate the predictive performance of the features in identifying postoperative respiratory depression.

**Results:**

Our findings indicated a similar performance of the proposed features to the conventional apnea–hypopnea index (AHI) for assessing sleep apnea severity, with average area under the curve ranging from 0.77 to 0.81. Notably, entropy and standard deviation of overnight SpO_2_ signal and below average burden showed comparable predictive capability to AHI but with minimal computational requirements and individuals’ burden, making them promising for screening purposes. Our sex-based analysis revealed that compared to entropy and standard deviation, below average burden exhibited higher sensitivity in detecting respiratory depression in women than men.

**Conclusion:**

This study underscores the potential of preoperative SpO_2_ features as alternative metrics to AHI in predicting postoperative respiratory.

## Background

Sleep apnea syndrome, a common respiratory disorder during sleep, is a major risk factor for postoperative respiratory depression [1–5]. Its prevalence is estimated to range from 9 to 38% in general population [6–8] and 18–67.6% among surgical patients [1, 9]. Sleep apnea syndrome is characterized by repeated interruptions in breathing during sleep, known as apneas (complete pauses in breathing) and hypopneas (partial reductions in airflow) [10]. These interruptions in breathing lead to intermittent hypoxemia (decreased blood oxyhemoglobin level), which is strongly associated with cardiovascular disorders such as hypertension and stroke [8]. The breathing irregularities of sleep apnea syndrome are exacerbated postoperatively [11] due to the respiratory-depressing effects of pain medications (mainly opioids). The consequence is the increased risk of cardiovascular complications [5], respiratory depression, cardiorespiratory arrest [1], and mortality [12]. Preoperative assessment of respiratory irregularities associated with sleep apnea syndrome is crucial to ensure optimal perioperative care and to prevent adverse outcomes.

Diagnosing and assessing the severity of sleep apnea syndrome have several challenges which limits its predictive power for postoperative respiratory depression. The gold standard technique for diagnosing sleep apnea syndrome is lab-polysomnography (PSG) where more than 10 signals, including brain activity (EEG), eye movement (EOG), muscle activity (EMG), heart rate (ECG), blood oxygenation (SpO_2_), nasal pressure (airflow), and respiratory efforts are recorded to assess respiration and apneas and hypopneas during sleep [13]. The severity of sleep apnea syndrome is assessed using the apnea–hypopnea index (AHI), which is the number of apneas and hypopneas per hour of sleep. PSG is uncomfortable, has long wait times, and requires expert knowledge [8, 14]. Moreover, the assessment of AHI through the analysis of multiple signals demands proficiency and entails a laborious and time-intensive process. Home sleep apnea testing (HSAT) is a recent alternative to PSG to evaluate sleep apnea syndrome at home using a subset of airflow, respiratory effort, and blood oxygenation signals, which sensors can be applied by the individual with minimal training [8]. HSAT has high false negatives [13] and screening questionnaires, such as STOP Bang, have low to moderate specificities [15, 16]. Thus, there is an unmet need for alternative metrics which are predictive of postoperative respiratory depression.

Hypoxemia, a common consequence of sleep apnea syndrome, can be assessed based on the changes in SpO_2_ levels which can be recorded continuously and affordably using a pulse oximeter from the finger. ODI, defined as the number of episodes with over 4% drops in SpO_2_ level per hour of sleep, and CT90 were shown to be predictive of postoperative adverse outcomes [17]. However, the performance of these metrics was limited to the area under the curve of 0.6 for ODI and 0.59 for CT90 [17]. To enable investigating more temporal and frequency domain characteristics of SpO_2_ signal, we have previously developed algorithms for automatic segmentation of SpO_2_ signal and extracting features from desaturation episodes. We validated our algorithm in a preliminary analysis using data from 50 individuals and we investigated the association of four features with AHI [18]. Our results showed that compared to AHI, measures of area under the curve of preoperative overnight desaturation episodes with ≥ 3% drops were more correlated with postoperative respiratory depression [18]. However, the sample size was limited, and we did not investigate the performance of the extracted measures to predict postoperative respiratory depression. Therefore, the primary aim of this study was to assess the effectiveness of SpO_2_ measures in predicting postoperative respiratory depression, building upon our prior research by enlarging both the sample size and the range of features examined.

## Results

In this retrospective analysis, we analyzed preoperative SpO_2_ signals from surgical patients to derive seven distinct features aimed at predicting postoperative respiratory depression (Table 1). The primary outcome of postoperative respiratory depression was defined as having at least one hypoxemia episode where SpO_2_ was less than 85% for more than 3 min [4]. Additionally, we examined the association of the extracted features with traditional assessment measures of sleep apnea syndrome, that is AHI (number of apneas and hypopneas per hour of sleep), total arousal index (the average of arousals per hour of sleep), and respiratory-related arousal index (the hourly average of arousals associated with apneas or hypopneas).

**Table 1:** Extracted SpO_2_ measures

Name	Descriptions
SpO_2_ ENT	Entropy of overnight SpO_2_ signal
SpO_2_ STD	Standard deviation of overnight SpO_2_ signal
BAB	Area under the overnight average of SpO_2_ divided by total sleep time in seconds (below average burden)
ODB AVG	Average of normalized overnight desaturation burdens^*^ of desaturation episodes
ODB STD	Standard deviation of normalized overnight desaturation burdens^*^ of desaturation episodes
ODB ENT	Entropy of normalized overnight desaturation burdens^*^ of desaturation episodes
NDB	Cumulative overnight desaturation burdens^*^ divided by the total sleep time in seconds (nocturnal desaturation burden)

^*^Desaturation burden: area under the curve of desaturation episodes with respect to the maximum SpO_2_ level within 100 s before SpO_2_ starts rising again

### Participants demographics

Out of 158 individuals whose data were included in the analysis, 27 individuals (17%) had postoperative respiratory depression. Characteristics of individuals with and without postoperative respiratory depression are presented in Table 2. While there were equal number of men and women in the study, the proportion of men and women differed between those with and without postoperative respiratory depression (*p* = 0.02). In individuals with postoperative respiratory depression, the proportion of women were significantly higher than men (70.37% women vs. 29.63% men, *p* = 0.032). No significant difference was observed between women and men among individuals without respiratory depression (45.80% women vs. 54.20% men, *p* = 0.336). Compared to individuals without respiratory depression, BMI, AHI, total arousal index, respiratory-related arousal index, and SpO_2_ measures (SpO_2_ STD, SpO_2_ ENT, BAB, ODB AVG, ODB STD, ODB ENT, NDB) were significantly higher in individuals with respiratory depression (*p* < 0.01 for all). Moreover, the prevalence of moderate to severe sleep apnea syndrome (AHI ≥ 15) was significantly higher in individuals with postoperative respiratory depression than those without respiratory depression (74.07% vs. 46.56%, *p* = 0.009).

**Table 2:** Patients’ demographics

	Total	No respiratory depression	Respiratory depression	p-value
Demographics				
N (%)	158 (100.0)	131 (82.91)	27 (17.09)	
Sex (N (%))				
Women	79 (50.00)	60 (45.80)	**19 (70.37)**^**1**^	**0.020**
Men	79 (50.00)	71 (54.20)	**8 (29.63)**^**1**^	
Age (years)	59.41 ± 11.26	59.34 ± 11.95	59.78 ± 7.01	lp
BMI (kg/m^2^)	30.28 ± 6.88	29.58 ± 6.38	33.66 ± 8.07	**0.007**
Comorbidities (N (%))				
Cardiorespiratory	93 (58.86)	73 (55.73)	20 (74.07)	0.078
Preoperative Sleep Features				
Apnea Hypopnea Index (hr^−1^)	20.20 ± 19.11	17.36 ± 16.58	33.95 ± 23.99	**0.001**
Total Arousal Index (hr^−1^)	20.38 ± 14.53	18.32 ± 12.32	30.38 ± 19.46	**0.002**
Respiratory-Related Arousal Index (hr^−1^)	12.48 ± 14.41	10.39 ± 11.89	22.66 ± 20.12	**0.004**
Moderate to Severe Sleep Apnea: N (%)	81 (51.27)	61 (46.56)	20 (74.07)	**0.009**
Preoperative Physiological Features				
Overnight SpO_2_				
Standard deviation (%)	1.93 ± 1.00	1.75 ± 0.72	2.81 ± 1.55	** < 0.001**
Entropy	1.85 ± 0.40	1.79 ± 0.35	2.14 ± 0.49	** < 0.001**
Overnight Desaturation Burdens				
Average	9.30 ± 3.08	8.77 ± 2.24	11.88 ± 4.83	**0.001**
Standard deviation	4.02 ± 2.40	3.65 ± 2.00	5.82 ± 3.23	** < 0.001**
Entropy	4.63 ± 0.96	4.53 ± 0.94	5.08 ± 0.95	**0.002**
Overall Nocturnal Desaturation Burden	2.91 ± 2.69	2.50 ± 1.94	4.87 ± 4.40	**0.005**
Below Average Burden	2.11 ± 1.09	1.92 ± 0.73	3.04 ± 1.82	** < 0.001**

**N:** Number, **BMI: **Body Mass Index, **Cardiorespiratory Comorbidities:** Arterial Hypertension, Coronary Artery Disease, Stroke, Angina, Myocardial Infarction, Heart Failure, Coronary Revascularization, Asthma, Chronic Obstructive Pulmonary Disease (COPD). ^1^p-value: 0.032. Continuous variables are presented with mean ± SD. Categorical variables are summarized as frequency (percentage). p-value of 0.05 is considered significant. p-value of test results where statistical power is ≥ 70% are presented, otherwise it is indicated as lp (low power)

Our sex-based analysis showed that in individuals with postoperative respiratory depression, the prevalence of moderate to severe sleep apnea syndrome was higher in women than men (12 vs. 8) while the severity was lower (average AHI: 40.63 h^−1^ vs. 50.06 h^−1^, average total arousal: 34.53 h^−1^ vs. 38.70 h^−1^, average respiratory-related arousal: 26.56 h^−1^ vs. 34.19 h^−1^). In individuals without postoperative respiratory depression, the prevalence and severity of moderate to severe sleep apnea syndrome was lower in women than men (prevalence: 23 in women vs. 38 in men, average AHI: 25.63 h^−1^ in women vs. 33.43 h^−1^ in men, average total arousal: 21.31 h^−1^ in women vs. 27.60 h^−1^ in men, average respiratory-related arousal: 13.65 h^−1^ in women vs. 20.62 h^−1^ in men).

**Fig. 1:**
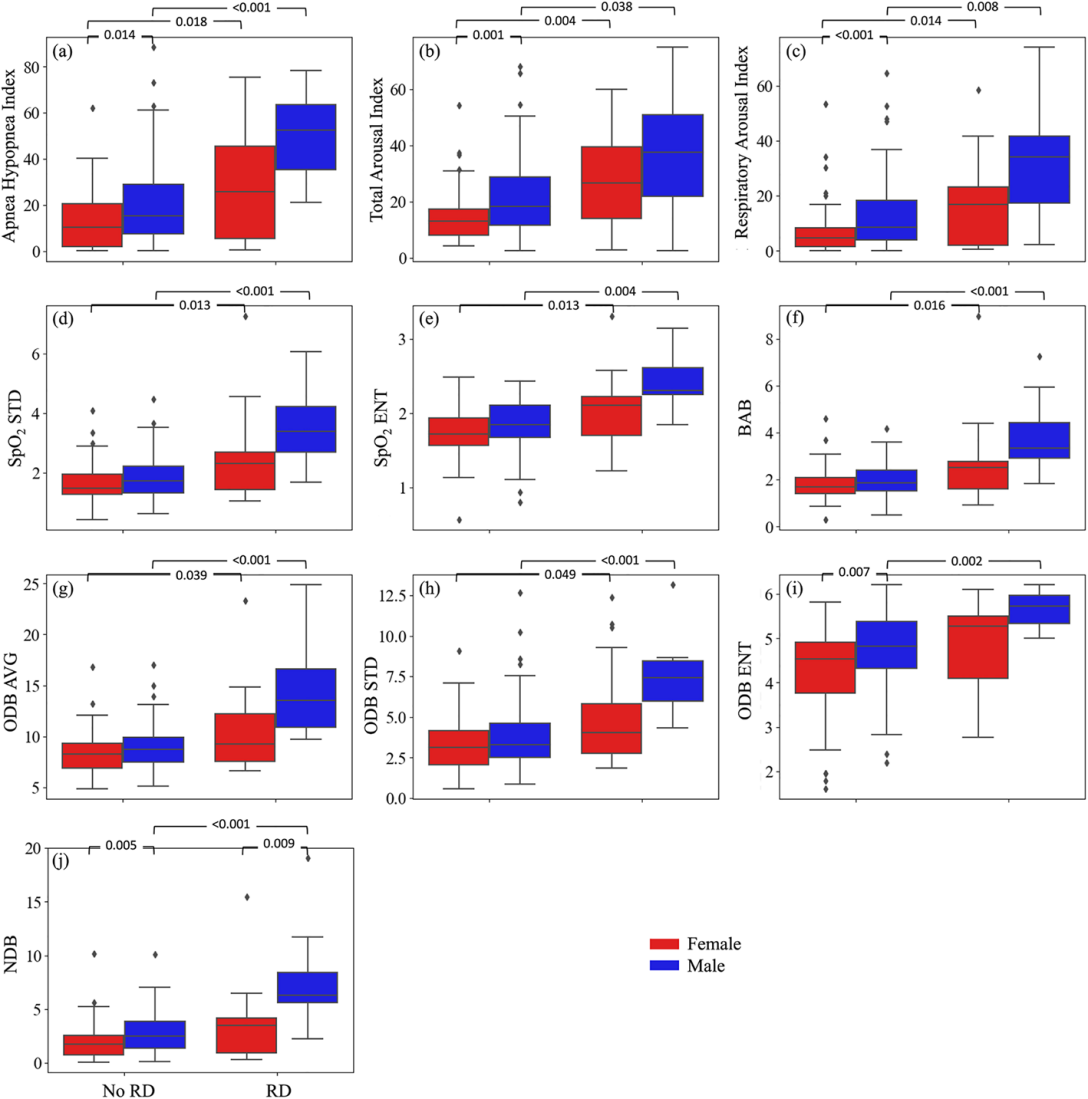
Sex-differences of sleep apnea and SpO_2_ measures in patients with and without postoperative respiratory depression. **a** apnea–hypopnea index (AHI), **b** total arousal index, **c** respiratory-related arousal index, **d** standard deviation of overnight SpO2 signal (SpO_2_ STD), **e** entropy of overnight SpO_2_ signal (SpO_2_ ENT), **f** below average burden (BAB), **g** average of normalized desaturation burdens of overnight desaturation episodes with ≥ 3% drops (ODB AVG), **h** standard deviation of normalized desaturation burdens of overnight desaturation episodes with ≥ 3% drops (ODB STD), **i** entropy of normalized desaturation burdens of overnight desaturation episodes with ≥ 3% drops (ODB ENT), **j** Overall nocturnal desaturation burden (NDB). RD: respiratory depression. Values show p-values

In both men and women, AHI, total arousal index, respiratory-related arousal index, and SpO_2_ measures, except for ODB ENT and NDB, were significantly higher in individuals with postoperative respiratory depression (*p* < 0.05 for all) (Fig. 1). The significance was stronger (p was lower) in men than women except for the total arousal index. In individuals with postoperative respiratory depression, AHI, total arousal index, respiratory-related arousal index, and SpO_2_ measures were higher in men than women. The difference was significant for NDB (7.99 ± 4.85 in men vs. 3.55 ± 3.43 in women, *p* = 0.009). In individuals without postoperative respiratory depression, AHI (20.97 ± 18.89 h^−1^ in men vs. 13.1 ± 12.35 h^−1^ in women, *p* = 0.014), total arousal index (21.43 ± 13.72 h^−1^ in men vs. 14.64 ± 9.40 h^−1^ in women, *p* = 0.001), respiratory-related arousal index (13.11 ± 13.30 h^−1^ in men vs. 7.17 ± 9.21 h^−1^ in women, *p* < 0.001), ODB ENT (4.75 ± 0.86 in men vs. 4.28 ± 0.97 in women, *p* = 0.007), and NDB (2.92 ± 2.03 in men vs. 2.01 ± 1.70 in women, *p* = 0.005) were significantly higher in men than women.

### Prediction of postoperative respiratory depression

**Fig. 2:**
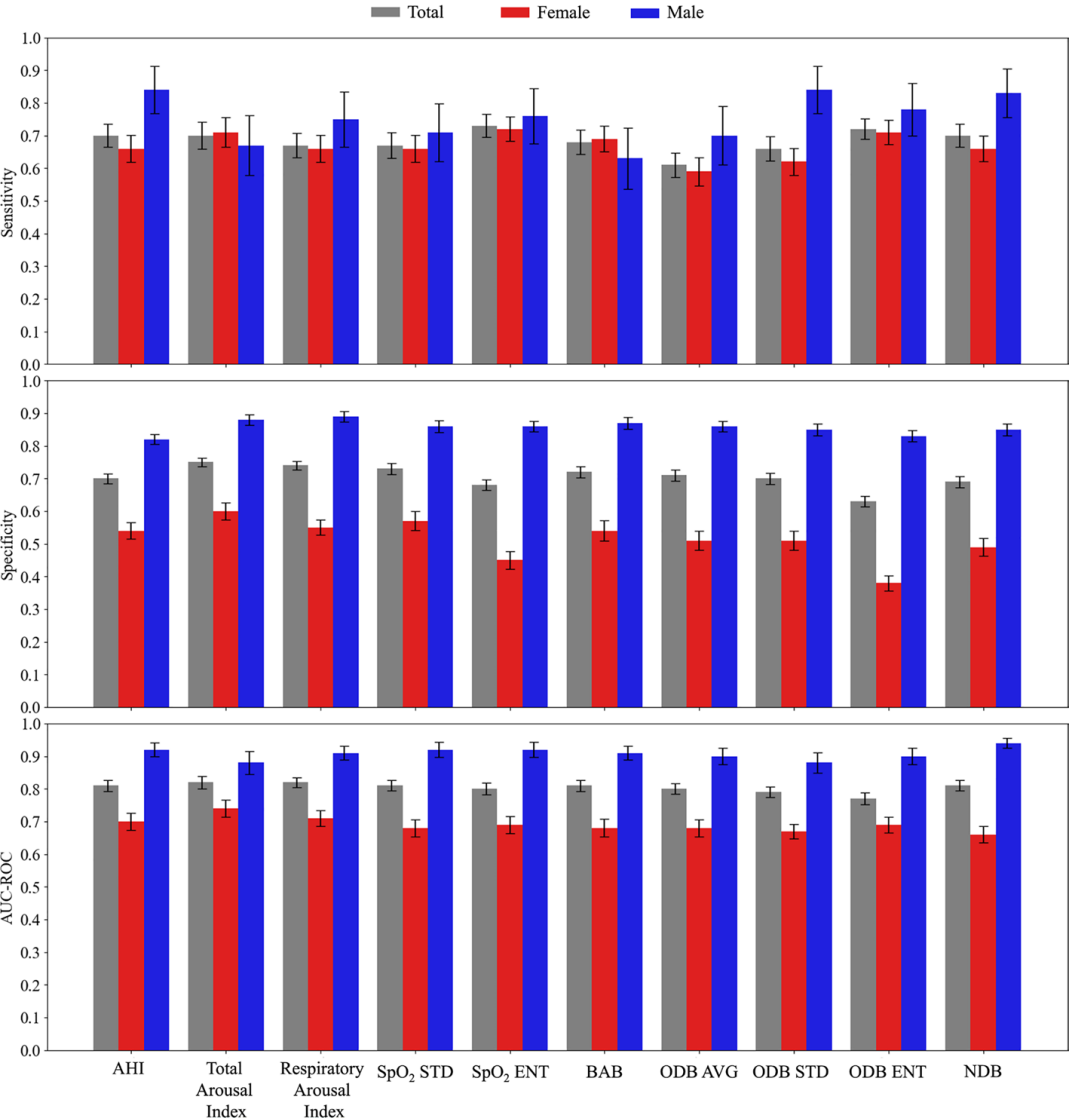
Performance of the sleep apnea and SpO_2_ measures in predicting postoperative respiratory depression. AHI: apnea–hypopnea index, SpO_2_ STD: standard deviation of overnight SpO_2_ signal, SpO_2_ ENT: entropy of overnight SpO_2_ signal, BAB: below average burden, ODB AVG: average of normalized desaturation burdens of overnight desaturation episodes with ≥ 3% drops, ODB STD: standard deviation of normalized desaturation burdens of overnight desaturation episodes with ≥ 3% drops, ODB ENT: entropy of normalized desaturation burdens of overnight desaturation episodes with ≥ 3% drops, NDB: Overall nocturnal desaturation burden. All models included sex, BMI, and pre-existing cardiorespiratory disorders (arterial hypertension, coronary artery disease, stroke, angina, myocardial infarction, heart failure, coronary revascularization, asthma, chronic obstructive pulmonary disease) to adjust for patients’ demographics. The error bars show the 95% confidence interval

Figure 2 presents the performance of SpO_2_ measures as well as AHI, total arousal index, and respiratory-related arousal index in predicting postoperative respiratory depression in total and between sexes, using logistic regression model. Our results showed that SpO_2_ STD, SpO_2_ ENT, BAB, ODB AVG, ODB STD, ODB ENT, and NDB were able to predict postoperative respiratory depression with average area under the receiver operating curve (AUC-ROC) of 0.81, 0.80, 0.81, 0.80, 0.79, 0.77, and 0.81, respectively. Except for ODB ENT, AUC-ROC of models with SpO_2_ measures were similar to the models with AHI, total arousal index, and respiratory-related arousal index. Specificity of the models with total (0.75) and respiratory-related (0.74) arousal index were significantly higher than AHI (0.70). Among SpO_2_ measures, SpO_2_ STD (0.73) and BAB (0.72) had highest specificities which were similar to the highest specificities of total arousal index and respiratory-related arousal index. The sensitivity of the models with SpO_2_ ENT (0.73) and ODB ENT (0.72) were higher than AHI (0.70), total arousal index (0.70), and respiratory arousal index (0.67). However, the differences were not significant. In summary, our sex-based analysis showed that in general the performance of the models is lower for women than men. Only in the models with total arousal index and BAB, average sensitivity was higher in women than men.

### Assessment of severity of sleep apnea syndrome

**Fig. 3:**
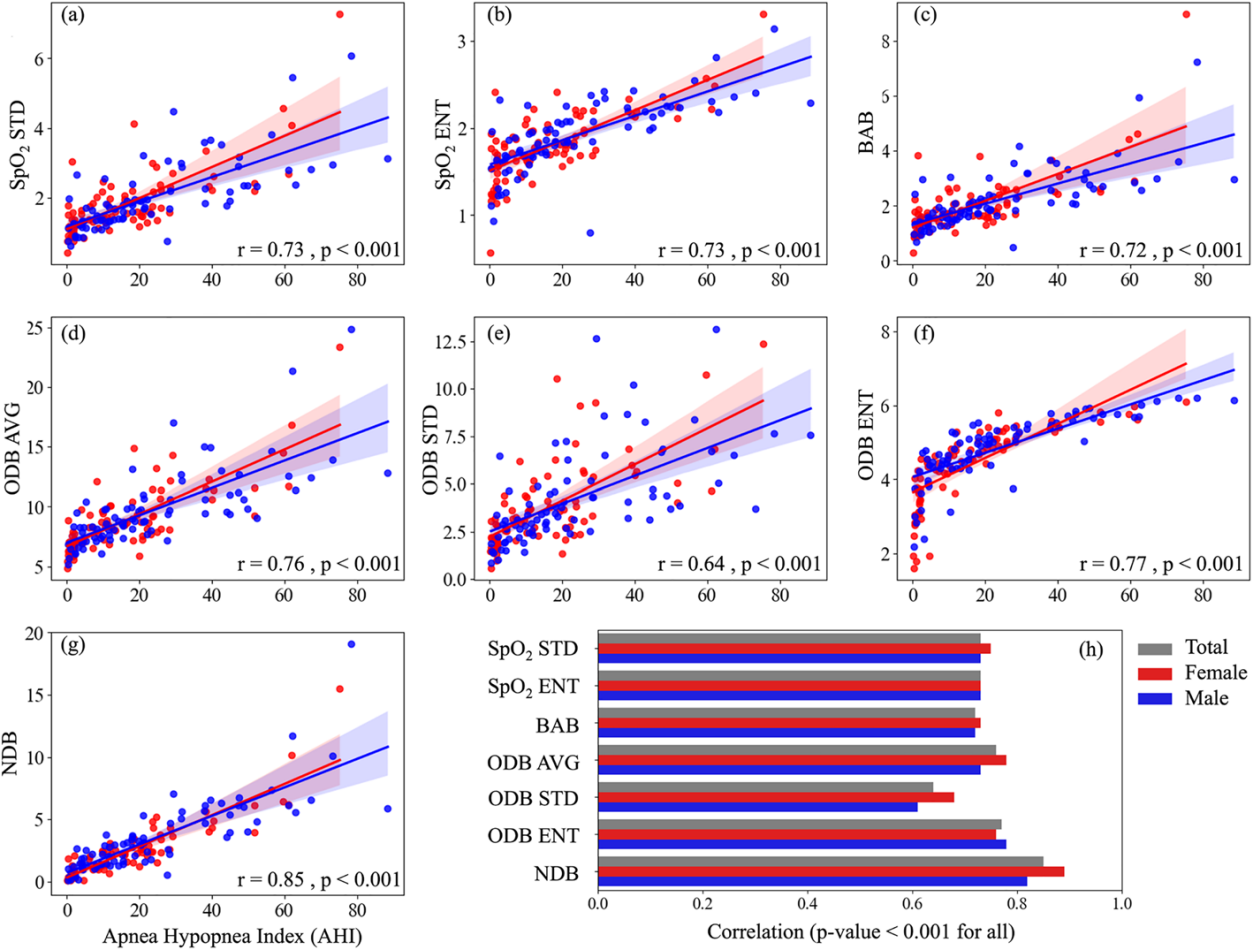
Correlation between apnea–hypopnea index (AHI) and SpO_2_ Measures. **a** standard deviation of overnight SpO2 signal (SpO_2_ STD), **b** entropy of overnight SpO_2_ signal (SpO_2_ ENT), **c** below average burden (BAB), **d** average of normalized desaturation burdens of overnight desaturation episodes with ≥ 3% drops (ODB AVG), **e** standard deviation of normalized desaturation burdens of overnight desaturation episodes with ≥ 3% drops (ODB STD), **f** entropy of normalized desaturation burdens of overnight desaturation episodes with ≥ 3% drops (ODB ENT), **g** Overall nocturnal desaturation burden (NDB). **h** correlation values

**Fig. 4:**
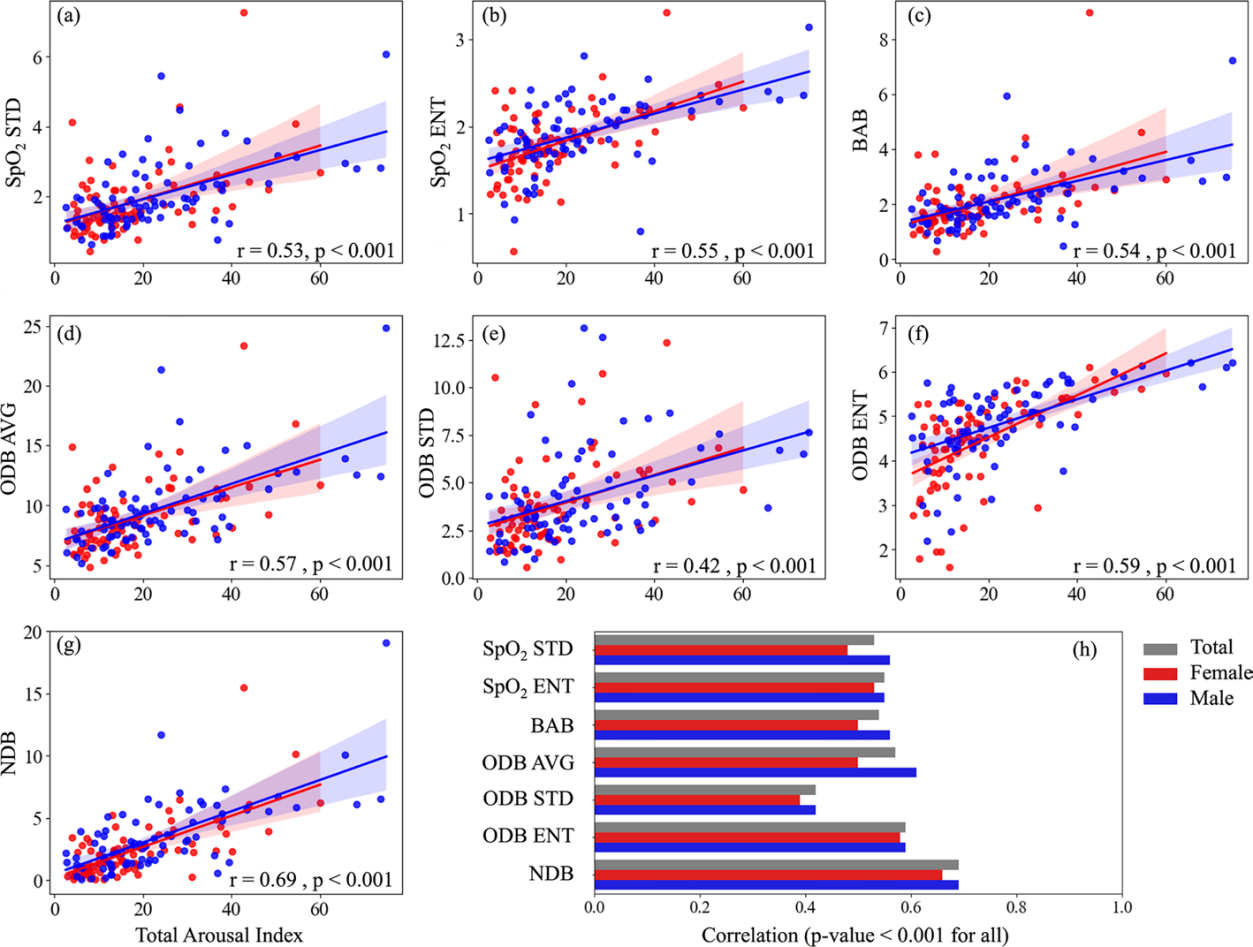
Correlation between total arousal index and SpO_2_ Measures. **a** standard deviation of overnight SpO_2_ signal (SpO_2_ STD), **b** entropy of overnight SpO_2_ signal (SpO_2_ ENT), **c** below average burden (BAB), **d** average of normalized desaturation burdens of overnight desaturation episodes with ≥ 3% drops (ODB AVG), **e** standard deviation of normalized desaturation burdens of overnight desaturation episodes with ≥ 3% drops (ODB STD), **f** entropy of normalized desaturation burdens of overnight desaturation episodes with ≥ 3% drops (ODB ENT), **g** Overall nocturnal desaturation burden (NDB).) correlation values

**Fig. 5:**
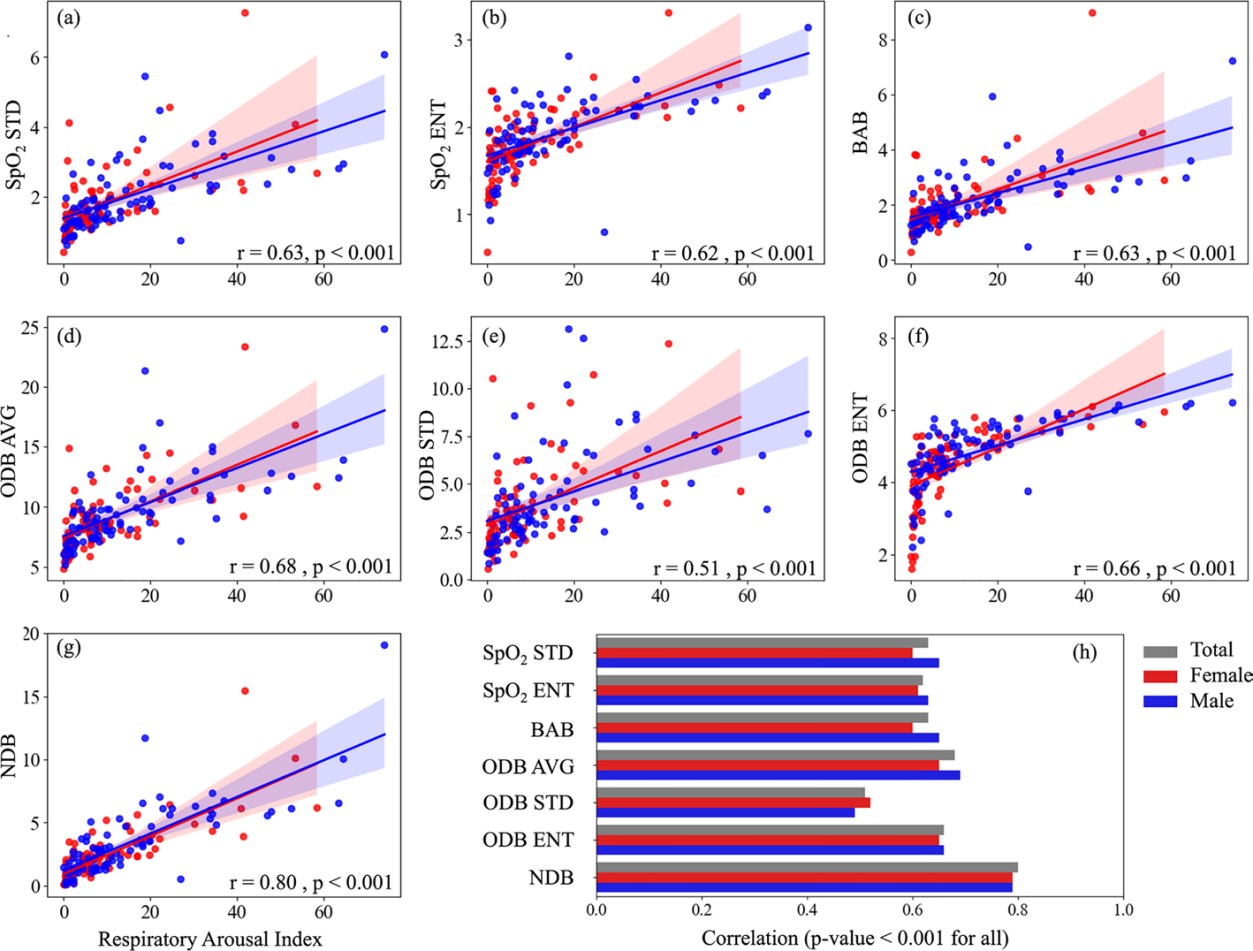
Correlation between respiratory-related arousal index and SpO_2_ Measures. **a** standard deviation of overnight SpO_2_ signal (SpO_2_ STD), **b** entropy of overnight SpO_2_ signal (SpO_2_ ENT), **c** below average burden (BAB), **d** average of normalized desaturation burdens of overnight desaturation episodes with ≥ 3% drops (ODB AVG), **e** standard deviation of normalized desaturation burdens of overnight desaturation episodes with ≥ 3% drops (ODB STD), **f** entropy of normalized desaturation burdens of overnight desaturation episodes with ≥ 3% drops (ODB ENT), **g** Overall nocturnal desaturation burden (NDB). **h** correlation values

The correlation between the SpO_2_ measures and AHI, total arousal index, and respiratory-related arousal index are presented in Figs. 3, 4, and 5. Our results indicated that SpO_2_ measures were significantly correlated with AHI, total arousal index, and respiratory-related arousal index (*p* < 0.001, for all). The correlation was stronger with AHI and respiratory-related arousal index than total arousal index and NDB had the highest correlation with AHI, total and respiratory-related arousal indices. AHI was correlated strongly with NDB (r = 0.85) and moderately with SpO_2_ STD (r = 0.73), SpO_2_ ENT (r = 0.73), BAB (r = 0.72), ODB AVG (r = 0.76), ODB STD (r = 0.64), and ODB ENT (r = 0.77). Total arousal index was correlated moderately with NDB (r = 0.69) and fairly with SpO_2_ STD (r = 0.53), SpO_2_ ENT (r = 0.55), BAB (r = 0.54), ODB AVG (r = 0.57), ODB STD (r = 0.42), and ODB ENT (r = 0.59). Respiratory-related arousal index was correlated strongly with NDB (r = 0.80), fairly with ODB STD (r = 0.51), and moderately with SpO_2_ STD (r = 0.63), SpO_2_ ENT (r = 0.62), BAB (r = 0.63), ODB AVG (r = 0.68), and ODB ENT (r = 0.66). Our sex-based analysis showed that except for the SpO_2_ ENT and ODB ENT, the correlation between the SpO_2_ measures with AHI was higher in women than men; the correlation between the SpO_2_ measures and total arousal index was lower in women than men; and except for NDB, the correlation between the SpO_2_ measures and respiratory-related arousal index was lower in women than men. Nonetheless, the difference was not significant.

## Discussion

In this paper, we presented measures of variation of preoperative overnight SpO_2_ and burden of desaturation episodes which were significantly correlated with the severity of sleep apnea syndrome and predictive of postoperative respiratory depression. Our results showed that the predictive power of the introduced measures was similar to those of AHI and arousal indices, with average AUC-ROC values ranging from 0.77 to 0.81. Notably, standard deviation, entropy, and below average burden of overnight oxygen saturation demonstrated predictive power comparable to that of traditional indices like AHI and arousal indices. Moreover, these measures can be computed with minimal computational resources, making them attractive options for screening purposes. Additionally, we observed that the below average burden of overnight SpO_2_ exhibited higher sensitivity in detecting respiratory depression among women compared to men, in contrast to entropy and standard deviation.

We defined respiratory depression based on its most common adverse outcome, that is hypoxemia [1]. Hypoxemia and subsequent hypercapnia significantly increase the risk of cardiorespiratory arrest [1]. Identifying individuals with postoperative hypoxemia will enable early interventions which can significantly alleviate the subsequent cardiorespiratory complications.

The main novelty of our proposed method is that unlike AHI and arousal indices which require recording of several signals, computation of our proposed SpO_2_ measures is only dependent on one signal. Monitoring of SpO_2_ offers a 3-fold advantage: 1) recording of SpO_2_ is facile, cost-effective, automatic, and uninterrupted with minimal training of the patient or the caregiver, whether in the comfort of one's home or within a medical facility; 2) SpO_2_ measures can be automatically extracted using the developed algorithms with minimal expert knowledge; 3) the algorithms can be embedded in pulse oximeter devices for automatic screening of patients. In particular, SpO_2_ STD and SpO_2_ ENT can be computed without a complex algorithm or expert knowledge. Thus, they can be used as simple alternatives to AHI in predicting postoperative respiratory depression.

The advantage of our SpO_2_ processing technique is that it detects desaturation episodes automatically based on signal derivatives. This eliminates the need for manual annotations to identify event start and end points, thus broadening its applicability beyond respiratory events that strictly adhere to > 10 s duration criteria for apneas and hypopneas. Furthermore, our prediction algorithm incorporates major risk factors for respiratory depression, such as sex, BMI, and pre-existing cardiorespiratory disorders, in the model to enhance the model’s clinical relevance and effectiveness.

Sleep apnea syndrome is a major risk factor for postoperative respiratory depression. Our results showed that more than 74% of patients with postoperative respiratory depression had moderate to severe sleep apnea syndrome. Due to the limitations of lab-PSG, HSAT, and questionnaires, sleep apnea syndrome is highly undiagnosed or overestimated, which may misguide the pre- and postoperative care of patients. Previously, it has been shown that ODI, CT90 [19], and sleep apnea specific hypoxic burden (SSHB) defined as the area under the curve of desaturation episodes of apneas and hypopneas [20], are significantly correlated with AHI (r = 0.89, 0.60, 0.7, respectively). Our results showed that the correlation between AHI and SpO_2_ STD, SpO_2_ ENT, ODB AVG, ODB STD, and NDB were higher than CT90 and SSHB. While the correlation of ODI and AHI was higher, the SpO_2_ measures extracted in this study were more predictive of postoperative respiratory depression (AUC-ROC: ≥ 0.79 for SpO_2_ measures vs. 0.6 for ODI).

Our sex-based analysis showed that the prevalence of moderate to severe sleep apnea syndrome was higher and the severity was lower in women than men in patients with postoperative respiratory depression. Seventy percent of patients with postoperative respiratory depression were women among whom 63% had moderate to severe sleep apnea syndrome. Since the ventilatory responses and cardiovascular consequences of hypoxemia is stronger in women than men [21, 22], women with sleep apnea syndrome are at higher risk of respiratory depression. We observed that there was a trend for stronger correlation between the SpO_2_ measures and AHI in women than men and stronger correlation between the SpO_2_ measures and arousal indices in men, albeit these differences were not significant. Moreover, our results showed that the models with total arousal index and BAB had higher sensitivity in predicting postoperative respiratory depression in women.

One of the limitations of our study is that we studied a limited number of features and examined their predictive power of postoperative respiratory depression separately. Further studies are required to investigate other features from SpO_2_ as well as a proper feature selection technique for the classification model. Moreover, it is important to note that in this study, only individuals without oxygen therapy or CPAP treatment were included in the analysis, as these interventions can significantly affect oxygen saturation levels. Recognizing that SpO_2_ is not reliable in assessing hypoxemia and respiratory depression in these individuals, future studies should explore other monitoring modalities such as respiratory rate, end-tidal CO_2_, or transcutaneous CO_2_ modalities to assess changes in respiration and the risk of respiratory depression in these individuals.

## Conclusion

Diagnosis and assessing the severity of sleep apnea syndrome is important in surgical patients for incorporating proper perioperative care to reduce the adverse outcomes. In this study, we proposed several measures of variations of preoperative overnight SpO_2_ and burden of desaturation episodes, which were highly correlated with the severity of sleep apnea syndrome and can predict postoperative respiratory depression with high sensitivity and specificity. These measures provide unique insights into the respiratory health of surgical patients, enabling tailored perioperative management strategies to mitigate complications. Notably, measures such as standard deviation, entropy, and below average burden of preoperative overnight SpO_2_, which require minimal computational resources, are favorable options for screening purposes. The developed algorithms facilitate automated SpO_2_ data extraction, allowing seamless integration into pulse oximeters or smartwatches for simplified and effortless patient screening. The predictive algorithm empowers physicians to readily identify high-risk respiratory depression cases, optimizing perioperative care.

## Methods

### Participants

We analyzed data from 235 surgical patients retrospectively. Participants were adults of 18 years and older who were of American Society of Anesthesiologists physical status I–IV undergoing non-cardiac procedures at a tertiary hospital in Toronto [11, 23]. Data from 77 individuals were excluded for the following reasons: 1) being on chronic opioids, 2) missing data of demographics, comorbidities, SpO_2_ recordings, or the severity of sleep apnea syndrome, 3) having oxygen therapy pre- or postoperatively or missing the information regarding oxygen therapy, 4) being on continuous positive airway pressure (CPAP) therapy pre- or postoperatively for sleep apnea syndrome or missing the information regarding CPAP therapy, 5) having SpO_2_ recordings of less than an hour, or 6) having SpO_2_ with more than 50% of signal being invalid (i.e. SpO_2_ = 0). The study was approved by the Research Ethics Board of the University Health Network (IRB: #17-5495).

### Measurements

Participants’ demographics (age, sex, body mass index [BMI]) and pre-existing cardiorespiratory comorbidities (arterial hypertension, coronary artery disease, stroke, angina, myocardial infarction, heart failure, coronary revascularization, asthma, and chronic obstructive pulmonary disease) were collected preoperatively. Participants underwent overnight sleep studies at home or in hospital preoperatively and on the third night after surgery [11, 23]. Sleep studies were performed using a HSAT (Embletta X100, Embla, Broomfield, CO), which included SpO_2_ recordings with a sampling frequency of 3Hz and a sampling resolution of 1%. Sleep studies were scored by a certified sleep technologist according to the guideline of American Academy of Sleep Medicine (2007) [24]. Apnea was defined as a decrease in airflow signal by over 90% and a duration of more than 10 s. Hypopnea was defined as reduction in the airflow signal by more than 50% and a duration of more than 10 s which was associated with either more than 3% oxygen desaturation or an arousal from sleep. Moderate to severe sleep apnea syndrome was defined as AHI ≥ 15 events per hour (hr^−1^) [25].

### Data processing

The processing pipeline included signal processing, feature extraction, prediction model development, and statistical analysis. Python 3.7 and JMP Pro 16 were used for data processing and statistical analysis, respectively.

*Signal processing:* included 1) preprocessing the signals to remove the noises related to setting up or removing the device, movement, or sampling resolution, including excluding the first and last 15 min of overnight signals and applying a median filter (window = 10 s) [18], 2) detecting desaturation episodes with ≥ 3% drops in preoperative SpO_2_ signals in 2 steps of finding the drops and the recovery phases [18], and 3) detecting respiratory depression episodes using a 85% threshold in postoperative SpO_2_ signals [18]. A drop phase initiated upon the onset of SpO_2_ decline, continuing till the minimum level. A recovery phase ensued as SpO_2_ began to increase, lasting until SpO_2_ returned to its initial level at the start of the desaturation. If desaturation episode did not recover to the SpO_2_ level at the start of the drop, the end of the desaturation was set as the time of maximum SpO_2_ within two minutes after the end of the drop.

*Feature extraction:* seven measures were extracted from preoperative SpO_2_ signals (Table 1): entropy (SpO_2_ ENT) and standard deviation (SpO_2_ STD) of overnight SpO_2_ signal; below average burden (BAB) which was defined as the area under the overnight average of SpO_2_ divided by total recording time in seconds; average (ODB AVG), standard deviation (ODB STD), and entropy (ODB ENT) of normalized overnight desaturation burdens; and overall nocturnal desaturation burden (NDB). Desaturation burden was defined as the area under the curve of desaturation episodes with respect to the maximum SpO_2_ level within 100 s before SpO_2_ starts rising again. Normalization was performed by dividing the burden to the duration of desaturation episode in seconds. NDB was defined as the cumulative overnight desaturation burdens divided by the total recording time in seconds.

*Prediction model development:* for each extracted measure, a logistic regression model (regularization: L2, optimization: LBFGS) was trained on 80% of the data (training set) for predicting postoperative respiratory depression (binary classification). Validation set, comprising 20% of the data, was used to assess performance of the model. We selected logistic regression as our prediction model to enable comparison with previously proposed metrics [17].

To mitigate the challenges associated with imbalanced datasets, we incorporated techniques like stratified sampling, class weights, and using appropriate evaluation metrics, such as AUC-ROC, sensitivity, and specificity, rather than accuracy. The training and validation sets were selected based on a stratified randomization process to make sure that they are matched in terms of sex, BMI, and the ratio of the respiratory depression cases. Since the age of participants with and without respiratory depression was similar, age was not included in the stratification process and model development. Class weights were added to the model to address the dataset imbalance during training. As for evaluation metrics, AUC-ROC is the probability curve of sensitivity with respect to 1-specificity for different classification thresholds and it presents the ability of the classifier in distinguishing the classes. Sensitivity is calculated as $\frac{TP}{TP+FN}$ and specificity is calculated as $\frac{TN}{TN+FP}$. TP is true positive, FN is false negative, TN is true negative, and FP is the false positive, considering 0.5 as the classification threshold.

To assess the robustness, generalizability, and reproducibility of the model, this process was repeated 100 times, and the average performance was reported. All models included sex, BMI, and pre-existing cardiorespiratory conditions (arterial hypertension, coronary artery disease, stroke, angina, myocardial infarction, heart failure, coronary revascularization, asthma, chronic obstructive pulmonary disease (COPD)) to adjust for individuals’ demographics. We opted for 100 runs over traditional cross-validation due to the imbalance in the data and the challenges associated with maintaining the same distribution of both classes in cross-validation in all runs. Our approach ensures robust evaluation, considering the imbalanced nature of the dataset, and minimizes bias in model performance estimation.

*Statistical analysis:* to compare the characteristics of patients with and without postoperative respiratory depression or within sexes, t-test or Mann–Whitney U test were used for numerical variables based on normality test. Chi-squared test was used for categorical variables. Pearson’s correlation was employed to investigate the relationship between the SpO_2_ measures and AHI, total arousal index, and respiratory-related arousal index. Correlations r  0.8, 0.6 ≤ r < 0.8, and 0.3 ≤ r < 0.6 are considered strong, moderate, and fair, respectively [26]. To investigate whether the correlations are significantly different between sexes, the 95% confidence interval of correlations were used. If the confidence intervals of correlations for men and women overlapped, the difference was assumed to be non-significant. Retrospective power analysis was performed and only the results where the statistical power was more than 70% were reported. *p*-value < 0.05 were considered statistically significant.

## Data Availability

The dataset of the study is not publicly available due to the restrictions of the ethics approval.

## Abbreviations

AHI: Apnea–hypopnea index
AUC-ROC: Area under the receiver operating curve
BAB: Below average burden
BMI: Body mass index
CPAP: Continuous positive airway pressure
CT90: Cumulative time percentage with SpO_2_ below 90%
ECG: Electrocardiogram
EEG: Electroencephalogram
EMG: Electromyography
EOG: Electrooculogram
FN: False negative
FP: False positive
HSAT: Home sleep apnea testing
NDB: Nocturnal desaturation burden
ODB AVG: Average of normalized overnight desaturation burdens
ODB STD: Standard deviation of overnight normalized desaturation burdens
ODB ENT: Entropy of normalized overnight desaturation burdens
ODI: Oxygen desaturation index
PSG: Polysomnography
SpO_2_: Oxyhemoglobin saturation signal
SpO_2_ ENT: Entropy of overnight SpO_2_ signal
SpO_2_ STD: Standard deviation of overnight SpO_2_ signal
SSHB: Sleep apnea specific hypoxic burden
TN: True negative
TP: True positive

## References

[1] Gupta K (2018). Risk factors for opioid-induced respiratory depression in surgical patients: a systematic review and meta-analyses. BMJ Open.

[2] Subramani Y, Nagappa M, Wong J, Patra J, Chung F (2017). Death or near-death in patients with obstructive sleep apnoea: a compendium of case reports of critical complications. BJA British J Anaesthesia.

[3] Weingarten TN (2015). Predictors of delayed postoperative respiratory depression assessed from naloxone administration. Anesth Analg.

[4] Khanna AK (2020). Prediction of opioid-induced respiratory depression on inpatient wards using continuous capnography and oximetry: an international prospective, observational trial. Anesth Analg.

[5] Pivetta B, Sun Y, Nagappa M, Chan M, Englesakis M, Chung F (2022). Postoperative outcomes in surgical patients with obstructive sleep apnoea diagnosed by sleep studies: a meta-analysis and trial sequential analysis. Anaesthesia.

[6] Peppard PE, Young T, Barnet JH, Palta M, Hagen EW, Hla KM (2013). Increased prevalence of sleep-disordered breathing in adults. Am J Epidemiol.

[7] Senaratna CV (2017). Prevalence of obstructive sleep apnea in the general population: a systematic review. Sleep Med Rev.

[8] Gottlieb DJ, Punjabi NM (2020). Diagnosis and management of obstructive sleep apnea: a review. JAMA.

[9] Chan MT (2019). Association of unrecognized obstructive sleep apnea with postoperative cardiovascular events in patients undergoing major noncardiac surgery. JAMA.

[10] Dempsey JA, Veasey SC, Morgan BJ, O'Donnell CP (2010). Pathophysiology of sleep apnea. Physiol Rev.

[11] Chung F, Liao P, Yegneswaran B, Shapiro CM, Kang W (2014). Postoperative changes in sleep-disordered breathing and sleep architecture in patients with obstructive sleep apnea. Anesthesiol J Am Soc Anesthesiol.

[12] Kessler ER, Shah M, Gruschkus SK, Raju A (2013). Cost and quality implications of opioid-based postsurgical pain control using administrative claims data from a large health system: opioid-related adverse events and their impact on clinical and economic outcomes. Pharmacother J Human Pharmacol Drug Ther.

[13] Rundo JV, Downey R (2019). "Polysomnography," in Handbook of clinical neurology.

[14] Van de Water AT, Holmes A, Hurley DA (2011). Objective measurements of sleep for non-laboratory settings as alternatives to polysomnography—a systematic review. J Sleep Res.

[15] Sutherland K, Phillips C, Cistulli P (2015). Efficacy versus effectiveness in the treatment of obstructive sleep apnea: CPAP and oral appliances. J Dent Sleep Med.

[16] Pivetta B (2021). Use and performance of the STOP-Bang questionnaire for obstructive sleep apnea screening across geographic regions: a systematic review and meta-analysis. JAMA Netw Open.

[17] Chung F, Zhou L, Liao P (2014). Parameters from preoperative overnight oximetry predict postoperative adverse events. Minerva Anestesiol.

[18] A Assadi, F Chung, M Hafezi, A Yadollahi. "Automatic derivation of nocturnal desaturation burden from oxyhemoglobin saturation signal." Presented at the IEEE Biomedical Circuits and Systems (BioCAS). Toronto. 2023.

[19] Chung F, Liao P, Elsaid H, Islam S, Shapiro CM, Sun Y (2012). Oxygen desaturation index from nocturnal oximetry: a sensitive and specific tool to detect sleep-disordered breathing in surgical patients. Anesth Analg.

[20] Azarbarzin A (2019). The hypoxic burden of sleep apnoea predicts cardiovascular disease-related mortality: the osteoporotic fractures in men study and the sleep heart health study. Eur Heart J.

[21] Kest B, Sarton E, Mogil JS, Dahan A, van Beek GM, Dahan A, Teppema L, Johannes H (1998). Opioid-induced analgesia and respiratory depression: sex differences. Physiology and pharmacology of cardio-respiratory control.

[22] Kendzerska T (2020). Cardiovascular consequences of obstructive sleep apnea in women: a historical cohort study. Sleep Med.

[23] Chung F, Liao P, Elsaid H, Shapiro CM, Kang W (2014). Factors associated with postoperative exacerbation of sleep-disordered breathing. Anesthesiol J Am Soc Anesthesiol.

[24] Iber C, Ancoli-Israel S, Chesson AL, Quan SF (2007). The AASM manual for the scoring of sleep and associated events: rules, terminology and technical specifications.

[25] Kapur VK (2017). Clinical practice guideline for diagnostic testing for adult obstructive sleep apnea: an American academy of sleep medicine clinical practice guideline. J Clin Sleep Med.

[26] Akoglu H (2018). User's guide to correlation coefficients. Turkish J Emergency Med.

